# Spontaneous Combined Lung and Bowel Herniation Due to Uncontrolled Asthma

**DOI:** 10.7759/cureus.34939

**Published:** 2023-02-13

**Authors:** Karol Avila-Castano, Wendy T Garzon-Siatoya, Alexei Gonzalez-Estrada

**Affiliations:** 1 Division of Pulmonary, Allergy, and Sleep Medicine, Department of Medicine, Mayo Clinic, Jacksonville, USA

**Keywords:** high intracavitary pressures, bowel herniation, lung herniation, cough, uncontrolled asthma

## Abstract

Spontaneous lung and bowel hernias are infrequent structural defects secondary to conditions that usually follow bouts of excessive straining. These two conditions have been individually well documented in the literature; however, there are seldom reports of this combination of defects. Here, we describe the case of a 69-year-old man diagnosed with combined spontaneous lung and colon herniation following an episode of severe coughing due to uncontrolled asthma. Early recognition and prompt treatment should be warranted to prevent complications.

## Introduction

Spontaneous lung and bowel hernias are extremely rare entities. The usual pathological processes responsible are sudden increases in intrathoracic and intraabdominal pressures secondary to excessive straining attributed to physical exercises, childbirth, sneezing, coughing, severe vomiting episodes, and other conditions, along with weakness in the intercostal and abdominal musculature [1,2]. In such cases, the herniation is usually partial, confined only to the anterior thoracic cavity or the peritoneal cavity, respectively.

There are documented cases of lung herniation due to uncontrolled asthma [2-4]; however, in this case report, we will describe a concomitant rupture of the thoracic and abdominal cavities with subsequent lung and colon herniation after a severe coughing episode in an asthmatic patient who was uncontrolled on medical treatment. Additionally, we will discuss the possible mechanisms underlying this uncommon clinical presentation as well as the relevant literature related to this case.

## Case presentation

A 69-year-old non-smoker obese (body mass index of 35 kg/m^2^) man with a past medical history of uncontrolled asthma on treatment with formoterol/budesonide 80/4.5 mcg presented to an outpatient clinic complaining of four weeks of worsening cough associated with progressive abdominal bruising. One week before the visit, the patient felt a "popping" sensation and dull pain in the middle of his chest immediately after a violent bout of coughing. He denied any previous surgeries, congenital anomalies, or trauma to his chest. He had a previous history of multiple admissions for asthma exacerbations, for which he was treated with systemic steroids.

**Figure 1:**
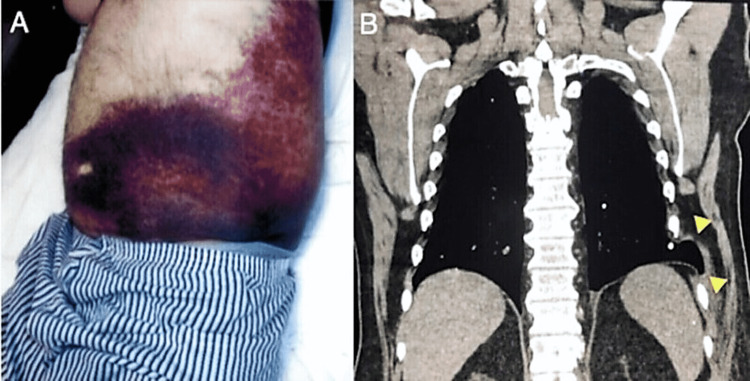
Clinical presentation of the patient (A) Ecchymosis on the left hypochondrium and the left iliac region, extending over the lower abdomen area. (B) Chest CT scan showing a left lateral hernia extending through the intercostal space between the eighth and ninth ribs (yellow arrowheads).

Physical examination showed an extensive ecchymosis between the left hypochondrium and the left iliac region, extending over the lower abdomen (Figure 1A). At palpation, the patient had a point of tenderness in the left mid-anterior thorax at the level of the eighth intercostal space. No points of tenderness were found in the abdomen, and the bowel sounds were normal. Vital signs were normal, and laboratory workup, including hemogram and coagulation studies, were unremarkable. The chest computed tomography (CT) scan showed a left-sided lung herniation extending through the intercostal space in between the eighth and ninth ribs (Figure 1B, yellow arrowheads), with a left-sided upper abdominal wall hernia extending over the anterior subcostal wall containing fat and the splenic flexure of the colon. The hernia had diaphragmatic and infra-diaphragmatic components.

The patient was referred for surgical evaluation and underwent thoracoabdominal surgery. Under general anesthesia, the surgeons performed an incision across the left costal margin. There was a disruption of the left costal margin between the eighth and ninth ribs with a hernia defect of 15 cm x 4 cm. The diaphragm and anterior abdominal wall muscles had stretched, revealing a defect through which both the lung and bowel were able to herniate. The hernias were reduced, and no alteration of the blood supply of the involved organs was noted. Repair of the defects was undertaken by re-approximating the ribs and performing an on-lay repair with prosthetic mesh at the eighth and ninth ribs. The patient did not have any complications and recovered uneventfully. Subsequently, the patient’s asthma was optimally controlled with a high dose of long-acting beta-agonist/corticosteroid inhaler without further episodes of cough or exacerbations.

## Discussion

Lung herniation is a very uncommon entity. Thoracic (60-80%) is the most frequent location for lung hernias, followed by cervical (15-35%) and diaphragmatic (2-5%) [3,5]. Etiologically, only 20% of lung hernias are congenital, and 80% are acquired. The majority of acquired hernias are traumatic (52%), followed by spontaneous (30%) and pathologic (18%) [3,5].

Spontaneous lung herniation usually results from an acute increase in intrathoracic pressure such as during protracted sneezing or coughing, musical instrument or glass blowing or heavy lifting [6]. Most of the documented cases of spontaneous lung hernia have been described in male, obese smokers with underlying chronic obstructive pulmonary disease [2].

Spontaneous bowel hernias are even rarer and also develop secondary to conditions that increased abdominal straining such as pregnancy, obesity, ascites and muscular weakness [7]. The most frequent location of a spontaneous bowel hernia is the posterior rectus sheath, which is located directly behind the rectus abdominis muscle. This type of hernia is caused by a weakening of the posterior rectus sheath, leading to a protrusion of the abdominal contents through the weakened tissue. Symptoms of a spontaneous bowel hernia can include abdominal pain, bulging of the abdomen, nausea, and vomiting. If the hernia is large enough, it can cause a strangulation of the bowel, leading to severe pain, abdominal distension, and even death [8].

In this particular case, the combination of uncontrolled asthma, chronic steroid use, and obesity have all been identified as contributing factors to the occurrence of these structural defects. In addition to the increased intracavitary pressures secondary to severe coughing and obesity, the prolonged use of steroids is suspected to have weakened the intercostal and intraabdominal muscles, which could further lead to the occurrence of herniation.

Physical examination and imaging tests such as CT scans, magnetic resonance, X-rays, and ultrasound are used to diagnose lung and bowel hernias [6,8]. A physical examination helps to determine if there is any tenderness in the thorax or abdomen, a lump or bulge, or other signs that could indicate the presence of a hernia. In both hernia types, the best diagnostic modality is a CT scan due to its high sensitivity to identify the presence of the herniated organ, the hernial sac, and an opening in the chest or abdominal wall [6,8]. It can also rule out potential complications such as lung or bowel tissue incarceration, obstruction, and strangulation.

The management of spontaneous combined lung and bowel herniation is surgical [9]. To avoid possible complications such as perforation, strangulation, and necrosis of the herniated organs, the thoracic and abdominal walls should be repaired in a timely manner. The surgical technique is chosen on a case-by-case basis, in which a simple suture using nonabsorbable sutures or synthetic mesh repair is necessary to reinforce the anterior abdominal wall, particularly close to the rib cage [9]. There is limited data regarding the recurrence rate of these combined hernias after treatment; however, some studies have found that for a first-time incisional hernia repair, the recurrence rate can be as high as 45%, and those repairs that use prosthetic material have fewer recurrences than when autogenous tissue is used. Furthermore, the history of wound healing disorders, some chronic comorbidities, and obesity, even in moderate cases (BMI of 28 and weight of 130% ideal body weight), increase the chance of recurrence. Most recurrent hernias become noticeable within the first three postoperative years [10,11].

## Conclusions

Spontaneous combined lung and bowel hernias are rare and potentially life-threatening conditions if missed or misdiagnosed. Delayed diagnosis can lead to incarceration and further enlargement of the defects. Clinicians should be aware of this uncommon condition, particularly when treating patients with uncontrolled asthma who present with unusual pain, bruising, and distortion of the chest and abdominal wall following a coughing episode. To ensure a favorable functional outcome, careful preoperative imaging, and an appropriate surgical approach to address the diaphragm, chest, and abdominal wall defects should be employed. Furthermore, optimal asthma management should be warranted to reduce the frequency and severity of exacerbations and consequently minimize the risk of occurrence of these types of structural defects that may require management by multiple specialists.
